# *CFH*, *C3* and *ARMS2* Are Significant Risk Loci for Susceptibility but Not for Disease Progression of Geographic Atrophy Due to AMD

**DOI:** 10.1371/journal.pone.0007418

**Published:** 2009-10-12

**Authors:** Hendrik P. N. Scholl, Monika Fleckenstein, Lars G. Fritsche, Steffen Schmitz-Valckenberg, Arno Göbel, Christine Adrion, Christine Herold, Claudia N. Keilhauer, Friederike Mackensen, Andreas Mößner, Daniel Pauleikhoff, Andreas W. A. Weinberger, Ulrich Mansmann, Frank G. Holz, Tim Becker, Bernhard H. F. Weber

**Affiliations:** 1 Department of Ophthalmology, University of Bonn, Bonn, Germany; 2 Wilmer Eye Institute, Johns Hopkins University School of Medicine, Baltimore, Maryland, United States of America; 3 Institute of Human Genetics, University of Regensburg, Regensburg, Germany; 4 Institute of Bioinformatics and Epidemiology, LMU Munich, Munich, Germany; 5 Institute for Medical Biometry, Informatics and Epidemiology, University of Bonn, Bonn, Germany; 6 Department of Ophthalmology, University of Wuerzburg, Wuerzburg, Germany; 7 Department of Ophthalmology, University of Heidelberg, Heidelberg, Germany; 8 Department of Ophthalmology, University of Leipzig, Leipzig, Germany; 9 Department of Ophthalmology, St. Franziskus Hospital, Muenster, Germany; 10 Department of Ophthalmology, RWTH Aachen University, Aachen, Germany; Ohio State University Medical Center, United States of America

## Abstract

**Background:**

Age-related macular degeneration (AMD) is a prevalent cause of blindness in Western societies. Variants in the genes encoding complement factor H (*CFH*), complement component 3 (*C3*) and age-related maculopathy susceptibility 2 (*ARMS2*) have repeatedly been shown to confer significant risks for AMD; however, their role in disease progression and thus their potential relevance for interventional therapeutic approaches remains unknown.

**Methodology/Principal Findings:**

Here, we analyzed association between variants in *CFH*, *C3* and *ARMS2* and disease progression of geographic atrophy (GA) due to AMD. A quantitative phenotype of disease progression was computed based on longitudinal observations by fundus autofluorescence imaging. In a subset of 99 cases with pure bilateral GA, variants in *CFH* (Y402H), *C3* (R102G), and *ARMS2* (A69S) are associated with disease (P = 1.6×10^−9^, 3.2×10^−3^, and P = 2.6×10^−12^, respectively) when compared to 612 unrelated healthy control individuals. In cases, median progression rate of GA over a mean follow-up period of 3.0 years was 1.61 mm^2^/year with high concordance between fellow eyes. No association between the progression rate and any of the genetic risk variants at the three loci was observed (P>0.13).

**Conclusions/Significance:**

This study confirms that variants at *CFH*, *C3*, and *ARMS2* confer significant risks for GA due to AMD. In contrast, our data indicate no association of these variants with disease progression which may have important implications for future treatment strategies. Other, as yet unknown susceptibilities may influence disease progression.

## Introduction

Age-related macular degeneration (AMD [MIM 603075]) is the leading cause of blindness in Western countries [1]. Two major forms of advanced AMD are responsible for vision loss. Geographic atrophy (GA) or “dry” AMD is characterized by an extensive loss of the choriocapillaris and the overlying retinal pigment epithelium, while the neovascular form or “wet” AMD develops due to invasion of neovascular complexes, a process known as choroidal neovascularization (CNV) [2]–[4]. Although constituting only 10–15% of all AMD cases, CNV accounts for almost 80% of AMD-related blindness. Nevertheless, with an increasingly aging population, blindness due to GA is predicted to become a significant socioeconomic burden in the near future [5]–[7]. To date, successful therapeutic intervention is available only for active CNV, while GA still remains untreatable [8], [9].

AMD is a complex condition with both genetic and environmental factors contributing to disease [2], [3], [10], [11]. Genetic variants at two chromosomal loci, 1q31 [12]–[15] and 10q26 [16], [17] confer major disease risks, together likely accounting for over 50% of cases. Chromosome 1q31 association is tightly linked to the complement factor H (CFH) gene suggesting an important role of inflammation and the alternative pathway of complement in AMD pathogenesis. This is further supported by strong association of AMD with variants in the complement component 3 (*C3* [MIM 120700]) gene [18], [19]. At 10q26, variants at two genes in strong linkage disequilibrium, namely age-related maculopathy susceptibility 2 (*ARMS2* [MIM 611313]) [17], [20] and HtrA serine peptidase 1 (*HTRA1* [MIM 602194]) [21], [22], were strongly linked to AMD susceptibility. Further studies will be needed however to clarify which of the two candidates plays the causal role in AMD pathology.

Going beyond cross-sectional association between AMD and genetic variants, Seddon et al. reported a significant association between the polymorphisms *CFH*-Y402H and *ARMS2*-A69S and progression from early or intermediate stages of AMD to the advanced forms [23]. It should be noted, however, that this study has not addressed the question of whether genetic variants are associated with disease progression once late AMD has developed. Nevertheless, it is the progression of the two late forms, GA and CNV, which is the most important variable with regard to future therapeutic intervention.

A quantitative measure of geographic atrophy progression represents the detection and quantification of atrophic areas on the fundus at different time points in order to calculate atrophy enlargement rates over time. The gold standard to define such areas has been fundus photography; however, the reproducibility for defining and measuring GA by fundus photography has been reported by several groups to be only moderate, particularly for smaller lesions [24]–[27]. An attractive alternative for non-invasive imaging of atrophy and its progression over time is fundus autofluorescence (FAF) imaging with the confocal scanning laser ophthalmoscope (cSLO). This method potentially allows direct visualization of loss of key cells in the disease process by tracing metabolic RPE changes. Therefore, areas of GA can now reliably be mapped facilitating absolute quantification of atrophic areas and their progression in the disease process [28]. Here, we used FAF imaging for phenotyping patients with GA in a longitudinal study, analyzed GA progression and correlated this biologically-based quantitative phenotype with genetic variants in the *CFH*, *C3*, and *ARMS2* genes.

## Results

Among the 619 participants of the FAM study cohort, 136 exhibited GA with no sign of CNV throughout subsequent follow-up examinations. Of these, 99 patients exhibited pure GA in both eyes throughout the study period and were included in the further analysis (for a comprehensive compilation of the phenotypic and genotypic data, see **Table S1**). Mean follow-up was 3.0 years (SD, 2.1; range, 6 months to 10 years). Mean number of measurements of GA area was 3.3 per eye (range, 2 to 12) and 3.5 per patient (range, 2 to 12). Mean age of patients was 71.8±7.4 years (range, 53–89 years) and 76.2±5.3 years (range, 65–97 years) for the controls (p<0.0001; t-test). Overall, 61% of the AMD patients and 62% of the controls were female (p = 0.837). Considering the most general measurement of smoking history as a binary “ever or never” variable, AMD patients were significantly more likely to smoke than controls (49% versus 13%; p<0.0001).

**Figure 1:**
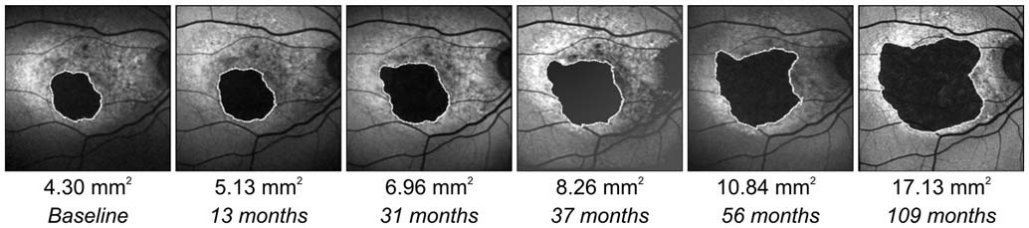
Progression of GA in a patient with age-related macular degeneration over a period of nine years. Atrophic areas are outlined in each image. Baseline GA area was 4.3 mm^2^; GA progression rate was 1.50 mm^2^/year based on 12 observations within nine years of clinical follow-up. GA growth rate of the fellow eye was 1.59 mm^2^/year. The patient's genotype was C/T for *CFH*-rs1061170, G/G for *ARMS2-*rs10490924, and C/G for *C3*-rs2230199.

In a case-control association analysis (N = 99 cases of pure bilateral GA; N = 612 controls), polymorphisms Y402H (rs1061170) in *CFH*, A69S (rs10490924) in *ARMS2* and R102G (rs2230199) in the *C3* gene were strongly associated with GA due to AMD (**Table 1**). In a case-only analysis (N = 99 GA cases), the median growth rate of GA was 1.61 mm^2^/year (interquartile range [IQR], 1.19 to 2.12; range, 0.26 to 3.45), which is very similar to our previous report [28]. A typical example of serial FAF images over a study period of 109 months is shown in **Figure 1**. Median GA area at baseline was 6.50 mm^2^ (IQR, 3.26 to 11.03; range, 0.05 to 34.6). As a measure of concordance of the GA progression rate between the right and left eye, the concordance correlation coefficient [29] was calculated at 0.726, 95% CI [0.587; 0.8297] indicating a high intra-individual symmetry of the GA progression rate in our cohort.

**Table 1:** SNP association in 99 AMD patients with bilateral pure GA versus 612 matched controls.

Gene (Marker)	Group	Genotypes (Frequency)			MAF	ATT P-value
*CFH* (rs1061170)		T/T	T/C	C/C		
	Cases	18 (0.184)	42 (0.429)	38 (0.388)	0.602	
	Controls	214 (0.350)	327 (0.535)	70 (0.115)	0·382	
	Odds Ratio [95% CI]	1 [Ref.]	1.53 [0.86,2.72]	6.45 [3.46,12.03]		1.63×10^−9^
*ARMS2* (rs10490924)		G/G	G/T	T/T		
	Cases	36 (0.364)	42 (0.424)	21 (0.212)	0.424	
	Controls	402 (0.658)	184 (0.301)	25 (0.041)	0.191	
	Odds Ratio [95% CI]	1 [Ref.]	2.55 [1.58,4.11]	9.38 [4.79,18.39]		2.58×10^−12^
*C3* (rs2230199)		G/G	G/C	C/C		
	Cases	54 (0.557)	35 (0.361)	8 (0.082)	0.263	
	Controls	394 (0.675)	176 (0.301)	14 (0.024)	0.175	
	Odds Ratio [95% CI]	1 [Ref.]	1.45 [0.92,2.30]	4.17 [1.67,10.40]		0.0032

Abbreviations: MAF, minor allele frequency; ATT, Armitage's trend test; CI, confidence interval.

**Figure 2:**
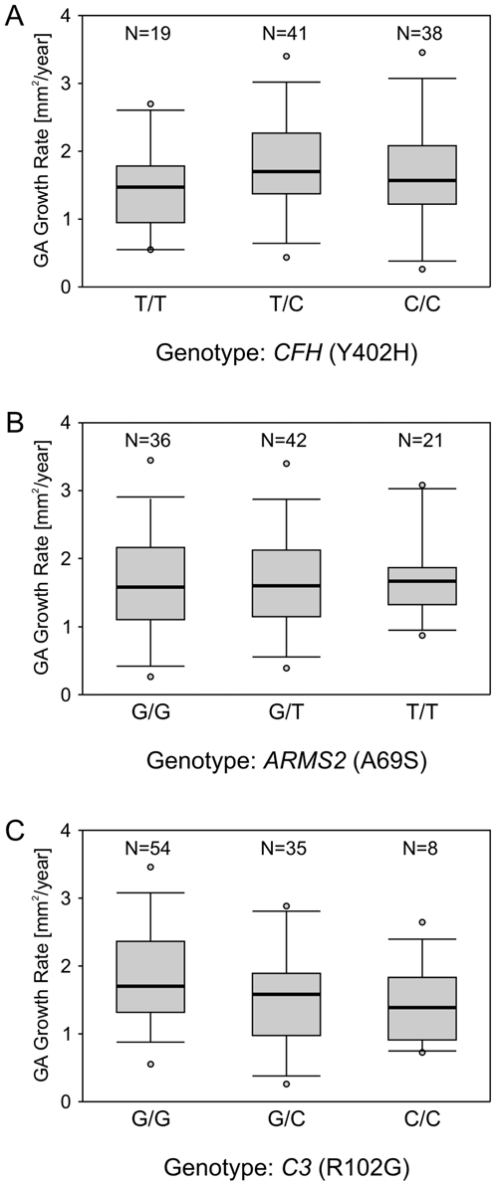
Growth rates of geographic atrophy for the subgroups of AMD patients grouped by genotypes for the three major risk alleles, *CFH* (A), *ARMS2* (B), and *C3* (C). Shown are the median (central bar), the 25^th^ and 75^th^ percentiles (grey box), the 5^th^ and 95^th^ percentiles (bars) and the minimum and maximum (small circles).

To visualize the distribution of GA growth rate for individual genotypes of *CFH* (Y402H), *ARMS2* (A69S), and *C3* (R102G), patients were grouped for genotypes of these three major risk genes (**Figure 2**). The association between the quantitative trait variable “GA progression” and SNP alleles in the *CFH*, *ARMS2* and *C3* genes was then analyzed (**Table 2**). Adjusting for age, smoking history and body mass index (BMI), and after a Sidak correction for multiple testing, none of the single-marker P values remained significant at alpha = 0.05. Similarly, there was no significant association between GA progression and the Y402H (rs1061170) and I62V (rs800292) haplotype of *CFH* (**Table 2**). Power calculations revealed a power of 80% (at alpha = 0.05) for detecting an additive allele effect of 0.278 mm^2^/year (Y402H, *CFH*), 0.275 mm^2^/year (A69V, *ARMS2*) and 0.292 mm^2^/year (R102G, *C3*), respectively. There was 95% power to detect an effect of less than 0.4 mm^2^/year for any of the three genetic risk factors. A detailed power calculation is given in **Table S2**.

**Table 2:** Association between the quantitative trait variable “geographic atrophy progression” and SNP alleles and haplotypes.

Gene (Marker)	Allele/Haplotype	Frequency (N = 99)	Additive Allele Effect in mm^2^/year (95% CI)^a^	P value^b^ (unadjusted)	P value^b^ (adjusted for age, smoking and BMI)	P value^b^ (corrected for multiple testing^c^)
*CFH* (rs800292*)*	G	172 (0.89)	Reference			
	A	22 (0.11)	0.0963 (−0.50, 0.70)			
				0.377	0.425	0.937
*CFH* (rs1061170)	T	78 (0.40)	Reference			
	C	118 (0.60)	0.2055 (−0.19, 0.60)			
				0.156	0.097	0.4
*CFH* (rs800292–rs1061170)	G–T	55 (0.29)	Reference			
	G–C	115 (0.60)	0.3048 (−0.15, 0.75)			
	A–T	22 (0.11)	0.3301 (−0.36, 1.02)			
				0.376^d^	0.204^d^	0.68
*ARMS2* (rs10490924)	G	114 (0.58)	Reference			
	T	84 (0.42)	0.0776 (−0.31;0.47)			
				0.233	0.376	0.905
*C3* (rs2230199)	G	143 (0.73)	Reference			
	C	51 (0.26)	−0.5141 (−0.98, −0.05)			
				1	1	1

a Progression rates relative to the reference allele/haplotype (non risk) which was set by definition to 0 mm^2^/year.

b Testing for a positive trend of progression (one-sided test).

c Sidak correction for five tests (4 SNPs and haplotype test).

d Two-sided test.

## Discussion

Here, we demonstrate a significant association between our study group with late AMD manifestations of pure GA and known variants at *CFH*, *C3*, and *ARMS2*. The odds ratios for heterozygotes and homozygotes for any of the three genes are highly consistent with previous reports [18], [19], [30]. Those reports typically included mixed study populations including both forms of late AMD, CNV and GA. This was also true for recent reports of R102G in *C3* as a significant risk variant for AMD where similar results were found for CNV and GA [18], [19]. DeWan and co-workers reported *HTRA1* as a risk gene for CNV due to AMD, implicating that polymorphisms in the *ARMS2-HTRA1* region are responsible for this specific phenotype of late AMD. This is a surprising notion since others, including our own study, did not find any difference in the association data between GA and CNV due to AMD [17]. Our current data now unambiguously demonstrate that the risk for GA is similarly associated with *ARMS2-HTRA1* variants and suggests that the association between the *CFH* and *ARMS2* polymorphisms is independent of disease evolution towards the atrophic or the neovascular form of AMD.

A significant original finding of this study is that none of the AMD-associated polymorphisms in *CFH*, C3 and *ARMS2* contribute to GA progression once GA has developed. It is of note that our analysis had good power to detect an additive allele effect of less than 0.3 mm^2^ of GA progression per year. A GA progression rate of 0.3 mm^2^ or less represents a relatively small effect with less than 1/5 of the mean effect in our study population. Moreover, it is clearly below the threshold of recent phase II studies with therapeutic intervention in GA (e.g. fenretinide trial; ClinicalTrials.gov identifier: NCT00429936) which aim to slow GA progression by at least 0.6 mm^2^/year.

Although the number of patients (N = 99; baseline, N = 619) may appear limited, the significance of this study mainly originates from its dependency on the major outcome variable which is a biologically based continuous variable. To put the size of our patient study group into perspective, the major population-based studies such as the Beaver Dam Eye Study as well as the major clinical trial sponsored by the National Eye Institute (e.g. Age-Related Eye Disease Study, AREDS) all include similarly limited numbers of longitudinally phenotyped patients with pure bilateral GA. Specifically, AREDS has 70 such patients (baseline, N = 3640) [31], while the Beaver Dam Eye Study has only 27 patients of at least 5-year follow-up (baseline, N = 4926; Ronald Klein, personal communication).

The strength of the current data is based on the phenotypic assessment of GA by cSLO-based FA imaging, semi-automated image analysis and complex statistics condensing longitudinal data into one continuous phenotypic variable. Although its accuracy may improve with the number of measurements over time, we have previously shown that GA progression is linear, which makes two consecutive measurements sufficient to estimate GA growth rate, although the number of longitudinal observations improves the accuracy of the estimated GA progression rate. [28], [32] An additional strong point in this study is the substantial number of patients with bilateral pure GA recruited through the multicenter FAM study.

Strong linkage disequilibrium (LD) across the *ARMS2-HTRA1* region makes it difficult to distinguish between *ARMS*2 [16], [17] and *HTRA1* [21], [22] as causal factors in AMD pathogenesis. We recently identified a deletion-insertion polymorphism in *ARMS2* (*372_815del443ins54) that is in almost perfect LD with the A69S variant and directly affects the stability of the transcript by removing the polyadenylation signal and inserts a 54-bp element known to mediate rapid mRNA turnover. [20] Immunohistochemistry has associated ARMS2 with mitochondria [33], specifically within the photoreceptor inner segments [20], suggesting that mechanisms of mitochondrial dysfunction may be involved in AMD pathogenesis.

Experimental [34] and clinical [35] data propose a key role of the major lipofuscin fluorophore A2-E in GA progression. Interestingly, A2-E biogenesis was recently linked to complement activation [36]. There is now compelling evidence that the complement system is involved in the pathogenesis of AMD and that the Y402H variant of *CFH* is associated with AMD susceptibility. Our data confirm this finding specifically for GA. Recently, we have detected systemic complement activation in AMD and found that markers of chronic complement activation were associated with the *CFH* risk haplotype (including the Y402H polymorphism). [37] However, since the Y402H variant of CFH confers similar risk of soft drusen and both forms of advanced AMD, it may contribute to the increased risk of advanced AMD largely or entirely through its impact on precursors of visually disabling AMD such as drusen. [38] Since polymorphisms in *CFH*, *C3*, and *ARMS2* are not associated with progression of GA and since our previous analysis did not detect a significant contribution of other characteristics (including smoking and BMI) [28] other modifying genetic factors may be involved.

Our findings may have strong implications for future efforts to design therapeutic interventions for AMD, although replication in an independent cohort and empirical treatment data are needed for further substantiation. Since variants within *CFH*, *C3*, and *ARMS2* confer risk for susceptibility of GA but appear unrelated to its progression, addressing the complement cascade or ARMS2-, and possibly HTRA1-associated pathways per se may not be promising strategies for alleviating disease progression once GA has developed. Instead, defining the exogenic and possibly the genetic factors responsible for GA progression could be crucial in helping those patients already suffering from advanced stages of the disease.

## Methods

### Ethics Statement

The study followed the tenets of the Declaration of Helsinki and was approved by the local Ethics Review Board at the University of Bonn. Informed written consent was obtained from each patient after explanation of the nature and possible consequences of the study.

### Cases and Controls

Patients with GA secondary to AMD were included from the longitudinal natural history arm of the multicenter FAM–Study (Fundus Autofluorescence in Age-Related Macular Degeneration; registration www.clinicaltrials.gov: NCT00393692). The study procedures have been previously reported. [28], [39] A total of 619 patients with dry (early or late) AMD in the study eye were enrolled (mean age, 73.9 years; mean follow-up, 35 months) and were recruited at six centres throughout Germany. Because of differences in genotype frequencies due to race and geographical origin, only white probands originating exclusively from Germany were included in the study.

Patients with uni- or multifocal GA and with clear vitreous to allow FAF imaging were included in the analysis. Exclusion criteria were a history of retinal surgery, laser photocoagulation and radiation therapy or other retinal diseases in the study eye such as diabetic retinopathy or hereditary retinal dystrophies. Fluorescein angiography was only performed if funduscopic signs were present indicative of neovascular AMD in addition to patches of GA. Such eyes were excluded from further analysis.

612 unrelated control individuals of German origin served as controls for case-control analysis to investigate the role of variants in CFH, C3 and ARMS2 for AMD susceptibility. [17] Each control subject underwent a single ophthalmic examination including visual acuity, slit lamp biomicroscopy and fundus ophthalmoscopy. Patients and controls underwent a general health interview protocol including a standardized smoking history.

### Phenotyping by FAF Imaging

FAF was measured using a confocal laser scanning ophthalmoscope (cSLO; Heidelberg Retina Angiograph, HRA classic and HRA 2, Heidelberg Engineering, Dossenheim, Germany), the optical and technical principles of which have been described previously. [40] An argon blue laser (HRA classic) or optically pumped solid state laser (HRA 2) were used for excitation (both 488 nm). The emitted light above 500 nm was detected with a barrier filter. FAF images were recorded in accordance with standard operating procedures (SOP) including focussing in the blue reflection mode, acquisition of a series of 30°×30° images (488 nm) and calculation of mean images after automated alignment in order to amplify signal to noise ratio with image analysis software (Heidelberg Eye Explorer, Heidelberg Engineering, Dossenheim, Germany). [41]

Only patient eyes with at least two follow-ups in a 6 month interval and with sufficient image quality to accurately determine the size of atrophy were included. To further improve on our previous analytical strategy [28], [32], serial FAF images of the same eye were aligned by the “4-point-alignment” function using Picture Window Pro 4.0.1.2 analysis software (Digital Light & Color, Cambridge, MA, USA). The total size of GA was measured in the processed FAF images by automated imaging analysis software that uses region-growing techniques to segment the areas of GA. [42] This procedure significantly reduced the estimated within-group error variance from 0.761 mm^2^/year, 95% CI [0.645; 0.898] previously [32] to 0.212 mm^2^/year, 95% CI [0.182; 0.246].

### Genotyping

Genomic DNA was extracted from peripheral blood leukocytes according to established protocols. Genotyping was done by TaqMan SNP Genotyping or by the matrix-assisted laser desorption/ionization time of flight (MALDI-TOF) mass spectrometry method (Sequenom, San Diego, CA, USA). TaqMan Pre-Designed SNP Genotyping Assays (Applied Biosystems, Foster City, CA, USA) were performed according to the manufacturer's instructions and were analyzed with a 7900HT Fast Real-Time PCR System (Applied Biosystems). All SNPs showed high genotyping quality with an average call rate of 98.5%.

### Statistical Analysis

Data management and statistical analysis were conducted using SAS Version 9.1.3 (Cary, NC, USA). The statistical analyses were carried out using software package R, Version 2.7.0 (http://www.r-project.org/), and the R-library “non-linear mixed effects” (NLME) was applied for hierarchical regression modelling. A linear mixed effects model was used to quantify GA growth. A detailed description of the longitudinal modelling process is provided elsewhere. [32], [43] The two-level-random effects model separates ocular-specific as well as patient-specific effects and helps to handle related observations. The mixed model methodology allows the study of variance components and fixed effects simultaneously. Using this two-level-random effects model, a single variable of GA progression rate was computed based on longitudinal observations of either one or both eyes for each patient studied. [32] This variable represents a continuous, biology-based quantitative phenotype and was used for association analysis with genetic data.

We used the case-control tool box from FAMHAP to perform quality control of the genetic data. [44] SNP genotypes in cases and controls showed no deviation from Hardy-Weinberg-Equilibrium at a level of alpha = 0.001. UNPHASED [45] was used to test for association between the quantitative trait variable “GA progression” and SNP alleles and haplotypes. We adopted the standard practice of genetic epidemiological studies to test in an allele-based manner when the underlying disease model is unknown. This has the advantage of reducing the number of degrees of freedom to 1. Although the resulting test is additive by nature, it is the most powerful test under a much broader range of true disease scenarios. The UNPHASED program provides P values as well as additive effect estimators for the effects of alleles/haplotypes on the quantitative trait. The reference alleles represent the respective major SNP alleles within Caucasian populations. The respective confidence intervals are provided as well. We also adjusted for the covariates age, BMI, and smoking, using the “modifiers” option of the UNPHASED software package. Posthoc power values for a t-test as an approximation of power for quantitative trait analysis for single SNPs were computed with the R software package, with the two groups of the t-test constituting the two alleles and the group sizes the allele counts. Covariates were not accounted for in the power calculation. Since the disease-associated allele is known, one-sided power values were computed.

## Supporting Information

Table S1 Clinical and molecular genetic findings in 99 patients with late-stage AMD
(0.27 MB DOC)

Table S2 Power values for alpha = 0.05 and additive allele effects of growth of geographic atrophy (n = 99)
(0.04 MB DOC)
