# Supplementary material for: CFH, C3 and ARMS2 Are Significant Risk Loci for Susceptibility but Not for Disease Progression of Geographic Atrophy Due to AMD
Source: PLoS One. 2009 Oct 12;4(10):e7418. doi: 10.1371/journal.pone.0007418 (PMC2756620; doi:10.1371/journal.pone.0007418)
Supplement: Table S1 — Clinical and molecular genetic findings in 99 patients with late-stage AMD (0.27 MB DOC) [file pone.0007418.s001.doc]

**Table S1** Clinical and molecular genetic findings in 99 patients with late-stage AMD (pure geographic athrophy)

| **Patient ID** | **Gender** | **Age**  **(yrs)** | **BMI*** | **Smoking** | **GA progression**  **rate OD**** | **GA progression**  **rate OS**** | **Patient-specific GA progression**  **rate** | ***CFH* rs1061170** | ***CFH* rs800292** | ***ARMS2***  **rs10490924** | ***C3* rs2230199** |
| --- | --- | --- | --- | --- | --- | --- | --- | --- | --- | --- | --- |
| 1017 | female | 72 | 23.8 | 1 | 1.54 | 2.04 | 1.71 | C/T | G/G | T/T | G/G |
| 1025 | female | 67 | 28.9 | 1 | no data | 0.73 | 0.87 | C/T | G/G | G/T | G/G |
| 1029 | female | 61 | 35.3 | 1 | 2.01 | 2.05 | 1.94 | T/C | A/G | G/G | C/C |
| 1033 | female | 78 | 24.5 | 1 | no data | 2.13 | 2.26 | C/T | G/G | T/G | G/G |
| 2001 | female | 73 | no data | no data | 1.20 | 1.58 | 1.48 | C/C | no data | G/G | no data |
| 2003 | male | 77 | 23.1 | 1 | no data | 1.41 | 1.42 | C/T | no data | T/G | no data |
| 2006 | female | 74 | no data | no data | 0.41 | 1.95 | 1.39 | C/C | G/G | T/T | G/G |
| 2007 | male | 53 | 34.0 | 0 | 1.50 | 1.59 | 1.61 | C/T | G/G | G/G | C/G |
| 2008 | female | 65 | 20.4 | 0 | 2.19 | 1.33 | 1.70 | C/C | G/G | G/G | C/G |
| 2012 | female | 80 | no data | no data | 1.01 | 1.03 | 1.09 | C/C | G/G | T/G | C/G |
| 2013 | female | 64 | 27.8 | 1 | 2.84 | no data | 2.60 | T/T | A/G | G/G | C/G |
| 2015 | female | 75 | 24.9 | 0 | 1.64 | 1.05 | 1.26 | C/C | G/G | T/T | G/G |
| 2017 | female | 72 | 26.7 | 1 | 1.89 | 1.41 | 1.70 | T/C | G/G | G/G | G/G |
| 2028 | female | 68 | 21.0 | 1 | 0.82 | no data | 0.90 | C/C | G/G | G/G | G/G |
| 2030 | male | 78 | 19.6 | 1 | 3.27 | 1.93 | 2.53 | C/C | G/G | T/G | G/G |
| 2053 | female | 68 | 26.0 | 1 | 0.97 | 0.68 | 0.99 | T/C | A/G | G/G | C/G |
| 2054 | male | 68 | 26.9 | 0 | no data | 1.51 | 1.37 | T/C | G/G | G/G | C/G |
| 2058 | male | 69 | 27.8 | 1 | 1.47 | no data | 1.47 | T/T | A/G | T/G | G/G |
| 2064 | female | 78 | 24.8 | 0 | no data | 2.28 | 2.01 | C/C | G/G | G/G | G/G |
| 2067 | female | 77 | 25.3 | 1 | 0.37 | 0.10 | 0.35 | C/C | G/G | G/G | C/G |
| 2073 | female | 67 | 31.2 | 0 | 2.10 | no data | 1.98 | C/C | G/G | T/G | C/G |
| 2074 | male | 74 | no data | no data | no data | 1.73 | 1.72 | T/C | G/G | G/G | C/G |
| 2080 | female | 81 | no data | no data | 1.68 | 1.28 | 1.44 | C/T | G/G | G/G | G/G |
| 2086 | female | 69 | 33.7 | 0 | 1.78 | 1.66 | 1.71 | T/C | G/G | T/T | C/G |
| 2087 | male | 66 | 22.1 | 1 | 1.72 | 2.02 | 1.82 | T/T | A/G | T/G | G/G |
| 2088 | male | 79 | 26.7 | 0 | 1.84 | 1.41 | 1.67 | T/C | G/G | G/G | C/G |
| 2089 | female | 71 | 21.3 | 0 | 1.78 | 1.90 | 1.80 | T/C | G/G | T/T | C/G |
| 2090 | female | 83 | 24.7 | 0 | 1.12 | 1.73 | 1.35 | T/T | G/G | G/G | G/G |
| 2091 | female | 53 | 28.3 | 0 | no data | 2.69 | 2.18 | T/C | G/G | T/G | C/G |
| 2109 | female | 66 | 30.1 | 1 | 1.64 | 2.27 | 1.88 | T/C | G/G | T/T | G/G |
| 2121 | female | 68 | 20.0 | 1 | 3.02 | no data | 2.51 | T/C | G/G | T/G | G/G |
| 2142 | male | 70 | 27.2 | 0 | 0.72 | 0.90 | 0.88 | T/C | G/G | G/G | G/G |
| 2144 | male | 59 | 29.0 | 1 | 0.93 | 0.76 | 0.87 | T/T | A/G | T/T | G/G |
| 2150 | male | 66 | 28.9 | 1 | 1.85 | 1.59 | 1.58 | C/C | G/G | T/G | G/G |
| 2159 | male | 75 | 26.4 | 1 | 1.09 | 1.23 | 1.21 | C/C | G/G | T/G | C/C |
| 2167 | female | 58 | 28.3 | 0 | 0.31 | no data | 0.55 | T/T | G/G | G/T | C/G |
| 2193 | female | 81 | 27.6 | 0 | 1.40 | 1.40 | 1.50 | C/C | G/G | T/T | C/G |
| 2197 | male | 70 | 23.7 | 1 | no data | 3.74 | 3.03 | T/C | G/G | T/T | G/G |
| 2205 | female | 72 | 22.4 | 1 | 0.31 | 0.27 | 0.39 | C/C | G/G | T/G | C/G |
| 2206 | female | 69 | 33.3 | 1 | 3.67 | 2.21 | 2.46 | T/C | G/G | G/G | G/G |
| 2207 | female | 80 | 23.6 | 0 | 3.20 | 3.78 | 3.40 | T/C | G/G | T/G | G/G |
| 2277 | female | 63 | 26.8 | 0 | no data | 0.47 | 0.95 | T/T | A/A | G/G | C/C |
| 2319 | male | 83 | 30.1 | 0 | 0.98 | 1.47 | 1.38 | T/C | G/G | T/T | G/G |
| 2323 | male | 66 | 25.8 | 0 | no data | 1.28 | 1.31 | T/C | A/G | T/G | G/G |
| 2378 | female | 83 | 27.9 | 0 | 1.80 | 1.58 | 1.67 | C/C | G/G | T/T | G/G |
| 2385 | male | 64 | 24.8 | 0 | 0.63 | 0.51 | 0.61 | T/C | G/G | G/G | C/G |
| 3007 | male | 68 | 27.7 | 0 | 1.66 | 1.59 | 1.78 | C/T | A/G | G/G | G/G |
| 3023 | male | 66 | 25.4 | 0 | 1.51 | no data | 1.45 | C/C | G/G | G/G | C/G |
| 3029 | male | 69 | no data | 1 | 1.38 | 0.87 | 1.20 | TC | A/G | T/T | G/G |
| 3037 | female | 67 | 25.8 | 0 | 2.95 | 3.30 | 2.80 | T/C | G/G | T/T | G/G |
| 3060 | male | 66 | no data | 1 | 1.33 | 1.40 | 1.31 | T/C | G/G | G/G | G/G |
| 3089 | female | 61 | no data | 1 | no data | 0.71 | 1.15 | T/T | G/G | T/G | G/G |
| 3091 | male | 67 | 23.7 | 1 | 1.08 | 1.34 | 1.44 | T/C | G/G | T/G | G/G |
| 3094 | female | 72 | 36.0 | 0 | 2.12 | no data | 1.87 | C/T | G/G | T/G | G/G |
| 3096 | female | 74 | 30.9 | 0 | 0.85 | 0.67 | 0.78 | T/T | G/G | G/G | C/C |
| 3098 | female | 75 | no data | 0 | 1.79 | 2.26 | 2.19 | C/C | G/G | G/G | G/G |
| 3099 | female | 62 | 23.9 | 0 | 1.53 | 1.81 | 1.67 | T/T | G/G | T/G | G/G |
| 3100 | female | 68 | 30.1 | 0 | 2.45 | 2.66 | 3.02 | T/C | A/G | G/G | G/G |
| 3101 | male | 67 | 29.7 | 1 | no data | 0.41 | 0.55 | T/T | A/G | T/G | G/G |
| 3103 | male | 75 | 23.7 | 1 | 3.17 | 2.44 | 2.78 | C/C | G/G | G/G | C/G |
| 3106 | male | 69 | 28.4 | 1 | 1.46 | 2.08 | 1.79 | C/C | G/G | T/G | C/C |
| 3107 | male | 59 | no data | 1 | 3.00 | 2.53 | 2.53 | T/C | A/G | G/G | G/G |
| 3114 | female | 73 | 27.5 | 1 | 0.11 | 0.21 | 0.26 | C/C | G/G | G/G | C/G |
| 3116 | female | 67 | 22.6 | 0 | 0.25 | no data | 0.44 | T/C | G/G | G/G | C/G |
| 3119 | female | 69 | 23.9 | 1 | 3.34 | no data | 3.45 | C/C | G/G | G/G | G/G |
| 3120 | female | 74 | 34.6 | 1 | 1.89 | 2.11 | 2.14 | C/T | A/G | T/G | G/G |
| 4002 | female | 84 | 29.6 | 0 | no data | 1.15 | 1.18 | C/C | G/G | T/G | C/G |
| 4003 | female | 72 | 20.7 | 0 | 3.20 | 3.07 | 2.83 | C/C | G/G | T/G | G/G |
| 4004 | female | 82 | 18.4 | 0 | 1.77 | 1.48 | 1.61 | C/C | G/G | T/G | C/G |
| 4009 | male | 74 | 25.8 | 0 | 0.76 | 1.96 | 1.49 | T/T | G/G | T/G | G/G |
| 4011 | male | 84 | 24.5 | 1 | 1.72 | no data | 1.58 | T/C | G/G | T/T | C/G |
| 4013 | female | 87 | 24.0 | 0 | 2.21 | 2.28 | 2.15 | T/T | A/G | G/G | G/G |
| 4017 | female | 68 | 29.3 | 0 | 1.01 | 0.82 | 0.95 | T/T | G/G | T/T | C/G |
| 4018 | female | 61 | 21.2 | 0 | 1.75 | 1.12 | 1.46 | C/C | G/G | G/G | C/G |
| 4019 | male | 89 | 22.5 | 1 | 1.62 | 1.98 | 1.89 | T/T | A/G | T/G | G/G |
| 5001 | male | 70 | no data | 1 | 1.75 | 1.50 | 1.56 | C/C | G/G | T/G | C/C |
| 5011 | male | 76 | 24.4 | 1 | 0.96 | 1.05 | 0.98 | no data | G/G | T/G | G/G |
| 5021 | female | 76 | no data | 0 | 1.06 | 0.34 | 0.72 | C/C | G/G | T/G | C/G |
| 5025 | female | 79 | 26.7 | 0 | 3.98 | 1.81 | 2.56 | C/T | G/G | T/G | G/G |
| 5030 | male | 84 | no data | 0 | 1.90 | 1.64 | 1.74 | T/T | A/G | T/G | G/G |
| 5037 | female | 68 | 32.3 | 0 | 3.44 | no data | 2.88 | C/C | G/G | T/G | C/G |
| 5038 | male | 81 | no data | no data | 2.70 | 1.79 | 2.10 | C/C | G/G | T/G | C/G |
| 5041 | male | 76 | no data | 0 | 1.36 | 0.61 | 0.97 | C/C | G/G | T/G | G/G |
| 5042 | female | 76 | 51.9 | 0 | 0.51 | 0.71 | 0.73 | T/C | G/G | T/G | C/C |
| 5045 | male | 79 | 26.3 | 1 | 1.49 | 1.78 | 1.69 | T/C | G/G | T/T | C/G |
| 5046 | female | 79 | no data | 1 | 2.66 | 2.76 | 2.64 | T/C | G/G | G/G | C/C |
| 5051 | male | 70 | 23.8 | 1 | 4.01 | 3.08 | 3.09 | C/C | G/G | T/T | G/G |
| 5052 | male | 70 | 36.3 | 1 | 1.96 | 2.14 | 2.04 | C/C | G/G | T/G | C/G |
| 5053 | female | 75 | 35.2 | 1 | 1.11 | 1.36 | 1.32 | C/C | G/G | T/T | G/G |
| 5055 | male | 66 | 26.0 | 1 | 1.02 | 1.03 | 1.02 | C/C | G/G | T/T | G/G |
| 6003 | female | 58 | 32.6 | 0 | 2.99 | 2.40 | 2.70 | T/T | A/A | T/T | C/G |
| 6006 | female | 78 | 25.4 | 1 | 3.15 | no data | 2.87 | C/C | G/G | G/G | C/G |
| 6007 | female | 65 | 26.0 | 1 | 1.49 | 1.19 | 1.55 | T/T | A/G | G/G | C/G |
| 6011 | male | 78 | 24.6 | 1 | 0.41 | 0.81 | 0.64 | C/T | G/G | T/G | C/G |
| 6015 | male | 71 | 27.0 | 1 | 2.54 | 3.75 | 3.08 | C/C | G/G | T/G | G/G |
| 6024 | female | 80 | 21.5 | 0 | 2.39 | 2.44 | 2.39 | T/C | G/G | T/G | G/G |
| 6025 | female | 80 | 28.3 | 0 | 2.02 | no data | 1.87 | C/C | G/G | T/T | G/G |
| 6027 | female | 68 | 22.6 | 0 | 2.06 | 0.35 | 1.14 | C/T | A/G | G/G | G/G |
| 6028 | female | 83 | 21.9 | 0 | 1.67 | 1.24 | 1.51 | C/C | G/G | T/G | G/G |

*** BMI: body mass index; ** Geographic Atrophy (GA) Progression Rate in mm^2^/year, OD** **(oculus dexter, right eye), OS (oculus sinister, left eye)**
