# Supplementary material for: CFH, C3 and ARMS2 Are Significant Risk Loci for Susceptibility but Not for Disease Progression of Geographic Atrophy Due to AMD
Source: PLoS One. 2009 Oct 12;4(10):e7418. doi: 10.1371/journal.pone.0007418 (PMC2756620; doi:10.1371/journal.pone.0007418)
Supplement: Table S2 — Power values for alpha = 0.05 and additive allele effects of growth of geographic atrophy (n = 99) (0.04 MB DOC) [file pone.0007418.s002.doc]

**Table S2.** Power values for alpha=0.05 and additive allele effects of growth of geographic atrophy (n=99)

| **SNP** | **Effect**  (mm^2^/year) | **Power** |
| --- | --- | --- |
| *CFH* (rs1061170) | 0.184 | 0.50 |
|  | 0.216 | 0.60 |
|  | 0.243 | 0.70 |
|  | 0.278 | 0.80 |
|  | 0.328 | 0.90 |
|  | 0.368 | 0.95 |
|  | 0.444 | 0.99 |
| *ARMS2* (rs10490924) | 0.182 | 0.50 |
|  | 0.210 | 0.60 |
|  | 0.240 | 0.70 |
|  | 0.275 | 0.80 |
|  | 0.324 | 0.90 |
|  | 0.364 | 0.95 |
|  | 0.440 | 0.99 |
| *C3* (rs2230199) | 0.193 | 0.50 |
|  | 0.223 | 0.60 |
|  | 0.256 | 0.70 |
|  | 0.292 | 0.80 |
|  | 0.344 | 0.90 |
|  | 0.387 | 0.95 |
|  | 0.467 | 0.99 |
